# Spatial response-code association for loudness but not brightness

**DOI:** 10.1177/03010066251361080

**Published:** 2025-08-04

**Authors:** Pui Leng Choon, Alexander Ludwig, Rolf Ulrich, Robert Carl Gunnar Johansson

**Affiliations:** Eberhard Karls Universität Tübingen, Tübingen, Germany; Eberhard Karls Universität Tübingen, Tübingen, Germany; 9188 Eberhard Karls Universität Tübingen, Tübingen, Germany; 9188 Eberhard Karls Universität Tübingen, Tübingen, Germany

**Keywords:** stimulus intensity, response code, lateral response set, generalized magnitude system, loudness, brightness

## Abstract

Cognitive associations between stimulus intensity and spatial response codes are thought to influence perceptual discrimination. We examined lateral response-set effects on auditory and visual intensity discrimination in a preregistered study with a large sample (*N* = 98). Participants responded to loud and bright stimuli using a button located to the left or right of the button used for soft and dim stimuli. In the auditory task, stimulus-response (SR)-mapping affected task-averaged error rates (ERs) but not task-averaged response times (RTs). However, loudness predicted response-side differences in both latency ( ${\text{RT}}_{\text{Left}}-{\text{RT}}_{\text{Right}}$ ) and accuracy ( ${\text{ER}}_{\text{Left}}-{\text{ER}}_{\text{Right}}$ ). By comparison, all tests of brightness discrimination supported the null or were inconclusive. Assessments of cross-modality correlations in SR-mapping effects were also inconclusive. These results replicate prior findings of lateral SR-mapping effects in auditory intensity discrimination and clarify inconsistencies in the visual domain. The lack of SR-mapping effects in brightness discrimination, along with inconclusive cross-modal correlations, challenges the notion of a common spatial processing mechanism for auditory and visual intensity comparison. If such a mechanism exists, its effects on visual judgments appear too subtle to be detected even in a large sample.

## Introduction

Conceptual associations between stimulus intensity and physical space are reflected in everyday language—for example, when we speak of “raising” or “turning down” the volume of a smartphone or speaker. This linguistic framing, where sound amplitude is referred to as “volume” or “level,” suggests that spatial cognition may aid how people think about and compare acoustic loudness. Comparable metaphors exist in the visual domain, such as when we “lower the brightness” of a screen, implying a similar spatial scaffolding of visual intensity perception. Beyond vertical metaphors, intensity-space mappings also appear along the horizontal axis, consistent with interface design conventions: users typically adjust loudness or brightness using horizontal sliders, where rightward movement increases intensity and leftward movement decreases it. This directional mapping aligns with well-documented spatial-conceptual associations, such as the left-to-right orientation of number lines (small to large; Dehaene et al., 1993; Wood et al., 2008) and timelines (past to future; Ulrich & Maienborn, 2010). The present study investigated whether lateral (left–right) intensity-space associations influence perceptual discrimination of auditory and visual stimuli. Of particular interest is whether spatial associations are shared across sensory modalities, or whether they operate in a modality-specific manner.

## Spatial Association for Loudness and Brightness

Behavioral research has shown that when people compare the intensity of two sequentially presented stimuli $S_{1}$ and $S_{2}$ , they are faster and more accurate when a “less intense” response ( $S_{2}<S_{1}$ ) entails a leftward button press, and a “more intense” response ( $S_{2}>S_{1}$ ) entails a rightward button press, as compared to when this mapping between stimulus and response (SR-mapping) is reversed. With respect to hearing, SR-mapping effects on auditory discrimination performance was demonstrated by Chang and Cho (2015) who leveraged both a loudness discrimination task (Experiment 1) and a timbre discrimination task (Experiment 2). When participants categorized loud signals with their right hand and soft signals with their left hand, performance was faster (shorter response times) and more accurate than when this mapping was reversed, suggesting a spatial association between acoustic loudness and lateral response set. This pattern emerged for both experiments so that the association between acoustic loudness and response side manifested not only for direct judgments of loudness, but also indirectly affected timbre judgments. Comparable results were reported by Hartmann and Mast (2016, Experiment 2C) and Fairhurst and Deroy (2017, Experiment 1), again suggesting that people associate sound intensity with direction in egocentric space.

In the visual domain, Ren et al. (2011, Experiment 3) reported evidence for an analogous spatial association between lateral response set and luminance level. In their paired discrimination task, two patches of light were presented sequentially: a standard stimulus of fixed brightness was followed by a variable comparison stimulus that was either dimmer or brighter relative to the standard. This study found that response times (RTs) were shorter when a dim stimulus entailed pressing the right response key and a bright stimulus entailed pressing the left key, indicating an SR-congruency effect between the response side and the magnitude of the imperative visual stimulus.^1^ Interestingly, this pattern is counter to what has been observed in the auditory domain, where faster responses are typically seen when loud stimuli are mapped to the right response key and soft stimuli to the left (Chang & Cho, 2015; Fairhurst & Deroy, 2017; Hartmann & Mast, 2016).

The directional reversal of lateral SR-mapping effects in brightness discrimination reported by Ren et al. (2011) may stem from a specific feature of their experimental design, namely the use of relatively dim targets presented against a middle gray background. This suggests that the critical factor influencing the SR association between brightness and response side might be contrast polarity—the relative difference between stimulus and background—rather than luminance per se. Supporting this interpretation, Fumarola et al. (2014, Experiment 1) used bright stimuli against a dark background (i.e., positive contrast polarity) and found an SR-mapping effect in the opposite and more typical direction: “brighter” responses were faster with the right response key, and “dimmer” responses were faster with the left. Furthermore, in a follow-up experiment (their Experiment 2), this effect persisted even when the hue of colored light patches was the target dimension and stimulus intensity was task-irrelevant, indicating that the SR association for brightness may operate automatically. Taken together, these findings suggest that contrast polarity plays a key role in shaping spatial associations for brightness, accounting for the reversed effect observed by Ren and colleagues.

Notably, SR-mapping effects persist even when the response axis is rotated from horizontal to vertical. For example, Bruzzi et al. (2017) found robust SR-mapping effects on both RTs and accuracy in a loudness discrimination task using vertically aligned response keys. Similarly, Koch et al. (2023, 2024) demonstrated vertical SR-mapping effects in auditory timbre discrimination tasks—even when loudness was a task-irrelevant dimension—suggesting an automatic spatial association. However, these findings contrast with those of Fairhurst and Deroy (2017), who reported no evidence of SR-mapping effects in a loudness discrimination task with response keys separated along the vertical dimension. This discrepancy highlights that the available evidence remains mixed, and might suggest potential task-specific factors that influence the emergence of spatial associations in rudimentary perceptual information processing tasks.

The ubiquity of SR-mapping effects across cognitive domains suggests that these phenomena extend beyond low-level sensory processing. Researchers have observed spatial compatibility effects not only for other perceptual features such as pitch (Lidji et al., 2007; Rusconi et al., 2006) and duration (Dalmaso et al., 2022; Ishihara et al., 2008; Mariconda et al., 2022), but also for abstract magnitudes such as numbers (Dehaene et al., 1993, 1990; Ito & Hatta, 2004) and even the heaviness of imagined objects (Dalmaso & Vicovaro, 2019; Vicovaro & Dalmaso, 2021). These converging findings have led to the proposal that a generalized brain system for processing both sensory information and spatial relations may subserve human reasoning about magnitudes across various cognitive domains, as briefly reviewed next.

## A Generalized System for Magnitude Processing

An open question is whether SR-mapping effects in intensity discrimination are modality-specific or reflect a more general association between stimulus intensity and spatial response coding. Multiple theoretical frameworks suggest that a generalized magnitude system subserves human reasoning across domains such as time, number, and space (Walsh, 2003; Winter et al., 2015). Support for this view comes from research in numerical cognition, particularly the SNARC-effect (Spatial-Numerical Association of Response Codes), which reveals that people more easily associate small numbers (e.g., 2) with left-hand responses and large numbers (e.g., 9) with right-hand responses than the reverse (Dehaene et al., 1990; Wood et al., 2008). This phenomenon is often interpreted as evidence for a “mental number line”—a spatial representation of the integers in ascending order of magnitude from left to right (1-2-3-4, etc.)—and implies that spatial coding and magnitude processing may draw on a common cognitive mechanism. Similar ideas are echoed by the polarity correspondence principle proposed by Proctor and Cho (2006), which holds that sensory attributes varying along a continuous “less–more” dimension (prothetic attributes; e.g., Stevens, 1957) are represented along the positive diagonal of a Cartesian mental plane.

The idea that a generalized magnitude system might underlie the spatial association between stimulus intensity and response codes draws support from a study by Cohen Kadosh and Henik (2006). They found that digits were judged faster and more accurately as numerically large when presented in a bright font compared to a dim font, whereas the reverse held for numerically small numbers. This brightness-number association was bidirectional: when the target dimension was switched from number to brightness, judgments were also more fluent when font luminance and digit cardinality were aligned (dim-small and bright-large) than when they were mismatched (dim-large and bright-small). The finding that brightness and numerical cardinality of printed digits are processed holistically led Cohen Kadosh and Henik (2006) to suggest that a generalized magnitude system in the parietal lobe might scaffold spatial reasoning, numerical cognition, and intensity processing. This idea has since gained empirical support from the cognitive neuroscience literature, as both number estimation and brightness estimation tasks are associated with increased blood flow in the right posterior intraparietal sulcus (Cohen et al., 2008; Sokolowski et al., 2017; Vogel et al., 2013).

In an interim summary, people are quicker and more accurate in intensity discrimination tasks when responding in a way that is spatially congruent with the intensity of the stimulus. However, it remains unclear whether SR-mapping effects operate similarly across different sensory modalities. Given theories suggesting a shared spatial mechanism underlying magnitude processing across sensory domains, this question warrants further investigation. The experiment reported below aimed to investigate the relationship between SR-mapping effects in auditory and visual intensity discrimination, with a focus on whether lateral response positioning influences perceptual judgments similarly in these two tasks.

## Experiment

This experiment investigated whether lateral SR-mapping effects in intensity discrimination are related across visual and auditory modalities—specifically, whether individuals who show strong mapping effects in vision also show them in audition. Participants completed two intensity discrimination tasks—one visual (brightness) and one auditory (loudness). The critical manipulation was the mapping between stimulus intensity (dim/bright and quiet/loud) and lateral response set (left/right key). Our procedure, analysis plan, and inferential statistical criteria were preregistered via the Open Science Framework.^2^

We anticipated that SR-mapping would affect stimulus-averaged mean correct RTs and error rates in both the visual and auditory tasks. Accordingly, we hypothesized that the regression slopes representing SR-mapping effects on mean RTs and error rates as a function of ordinal stimulus value would be significantly different from zero for both tasks. Most pertinent, we also hypothesized that the magnitude of the SR-mapping effects—measured as changes in stimulus-averaged mean RTs and error rates between SR-mappings—would correlate across tasks: participants who exhibit strong spatial mapping effects in one modality should tend to show similar effects in the other.

## Methods

### Participants and Sample Size

One hundred participants with a mean age of 33 years (age range: 18–66, 36 females and 92 right-handed) were recruited from a participant pool of native German speakers currently residing within Germany via the online platform Prolific.^3^ Participants were reimbursed with 8€ for partaking in a single, 45-minute session. Smartphone and tablet users were excluded, allowing only PC users to be sampled. All reported normal or corrected-to-normal seeing and normal hearing and provided informed consent. The target sample size was determined based on available resources for testing.

### Stimuli and Apparatus

The experiment was developed using PsychoPy (Peirce et al., 2019) and translated into JavaScript for online implementation via the launch platform Pavlovia.^4^ Visual stimuli were monochromatic (gray scale) squares measuring 0.2×0.2 height units relative to screen size. The standard stimulus in the brightness discrimination task was centered at a luminance value of [0, 0, 0] in RGB-space. The six visual comparison stimuli ranged from dark gray ([ −0.75,−0.75,−0.75 ]) to light gray ([0.75, 0.75, 0.75]) in RGB increments of 0.25. Auditory stimuli were 220 Hz pure tones. The standard tone was presented at a normalized amplitude of 0.5, with comparison tones spaced in increments of 0.04. Participants indicated their responses using the “E” and “P” keys on their computer keyboard, chosen for their clear spatial separation and consistent layout across common European keyboard formats (e.g., QWERTZ, QWERTY, and AZERTY).

### Procedure and Design

Participants were greeted with written instructions on the monitor underscoring the importance of responding fast and accurately. New trials were signaled by a faint fixation cross presented in the center of the monitor for 500 ms, followed by a blank 500 ms foreperiod. Then, the standard stimulus was presented for 500 ms followed by an empty 800 ms interstimulus interval. One of six possible comparison stimuli was then presented for 500 ms or until the response of the participant had been registered. If the response was incorrect, the German word for “Error” (“Fehler”) was presented in red ink for 500 ms, followed by a 500 ms intertrial interval. If the response was correct, the intertrial interval was 1,000 ms. Participants performed the experiment in a setting of their choice.

The critical experimental manipulation involved mapping the intensity of the comparison stimulus (weaker or stronger relative to the standard) to the lateral position of the corresponding response key (left or right). In SR-congruent blocks, participants were to press the right response key when the comparison was louder or brighter than the standard, and the left response key when the comparison was softer or dimmer. Correspondingly, participants were to press the left response key when the comparison was louder/brighter and the right response key when the comparison was softer/dimmer in the SR-incongruent condition. The 2 task modalities (visual or auditory) × 2 SR mappings (congruent or incongruent) were manipulated factorially, resulting in four block types (visual-congruent, visual-incongruent, auditory-congruent, and auditory-incongruent). Each comparison stimulus was cycled through randomly 20 times per block and each block was presented once, yielding 120 standard-comparison sequences per condition. Experimental blocks were always preceded by practice blocks comprising 24 trials, resulting in a total of 4×(120+24)=576 experimental and practice trials per participant. Participants were allowed to take self-paced rests after each practice and experimental block. Block presentation order was counterbalanced across participants.

### Data Analysis

Participants exhibiting <75% accuracy in either task (loudness discrimination or brightness discrimination) were removed from all analyses. Two participants were excluded for low accuracy (one performing at chance level), yielding a final sample size of *N* = 98. Individual outlier responses shorter than 150 ms or longer than 2,000 ms were also discarded. Few responses were too fast (0.1%) or too slow (1.9%). Formal data analysis proceeded through a mixture of preregistered hypothesis testing using the Bayes factor (BF) approach (Lee & Wagenmakers, 2014) complemented by Bayesian parameter estimation (Kruschke, 2014) using Markov Chain Monte Carlo (MCMC) sampling.

In the first step of analysis, main effects of SR-mapping (congruent vs. incongruent) on ERs and mean correct RTs were tested for each task modality separately using paired Bayesian *t*-tests. Incorrect responses were discarded from the analysis of RTs. Next, we calculated difference scores in ERs ( ${\text{ER}}_{\text{Left}}-{\text{ER}}_{\text{Right}}$ ) and mean correct RTs ( ${\text{RT}}_{\text{Left}}-{\text{RT}}_{\text{Right}}$ ) for each of six ordinal levels of stimulus intensity (1, 2, 3 and 5, 6, 7 with the nested standard stimulus coded as 4). The association between difference scores and ordinal stimulus intensity was tested for both task modalities using Bayesian correlation tests. We also regressed difference scores against ordinal stimulus intensity for both tasks separately using Bayesian linear regression to compute regression slopes and intercepts for graphical presentation. Finally, we correlated and regressed stimulus-averaged difference scores in both ERs and mean RTs between the loudness and brightness discrimination tasks to determine if the magnitude of SR-mapping effects was associated across task modalities.

All statistical modeling was conducted in R software (R Core Team, 2021). Inferential analyses relied on the default priors provided by the *BayesFactor* package (Morey & Rouder, 2024). Bayesian parameter estimation was conducted using Gibbs sampling with 10,000 MCMC steps, reported below in terms of correlation coefficients ( ρ ), nonstandardized effect sizes, and 95% highest density intervals (95% HDIs). Parameter estimates for all linear regression models are portrayed graphically in terms of their median posterior estimates and 95% HDIs. The threshold of statistical significance was BF=3 for all confirmatory tests (equivalent to $\text{BF}=\frac{1}{3}$ for establishing the null hypothesis).

## Results

**Figure 1.**
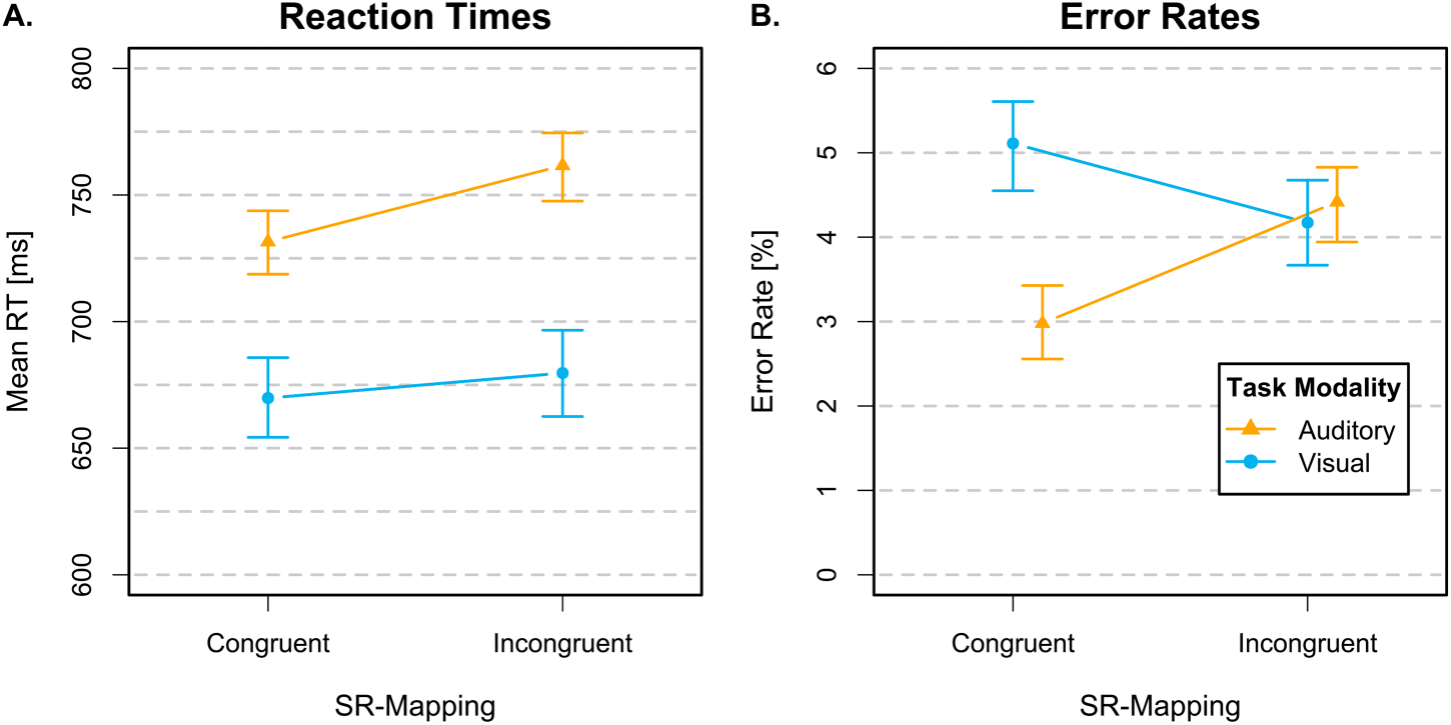
Task-averaged mean correct RTs (Panel A) and error rates (Panel B) depicted in terms of their median posterior estimates, separately as blue circles for the brightness discrimination task and orange triangles for the loudness discrimination task. Error bars represent 95% HDIs. Note that the HDIs are calculated based on mean-centered data following Cousineau (2005) and Loftus and Masson (1994). Abbreviations: RT = response time; HDIs = highest density intervals.

**Figure 2.**
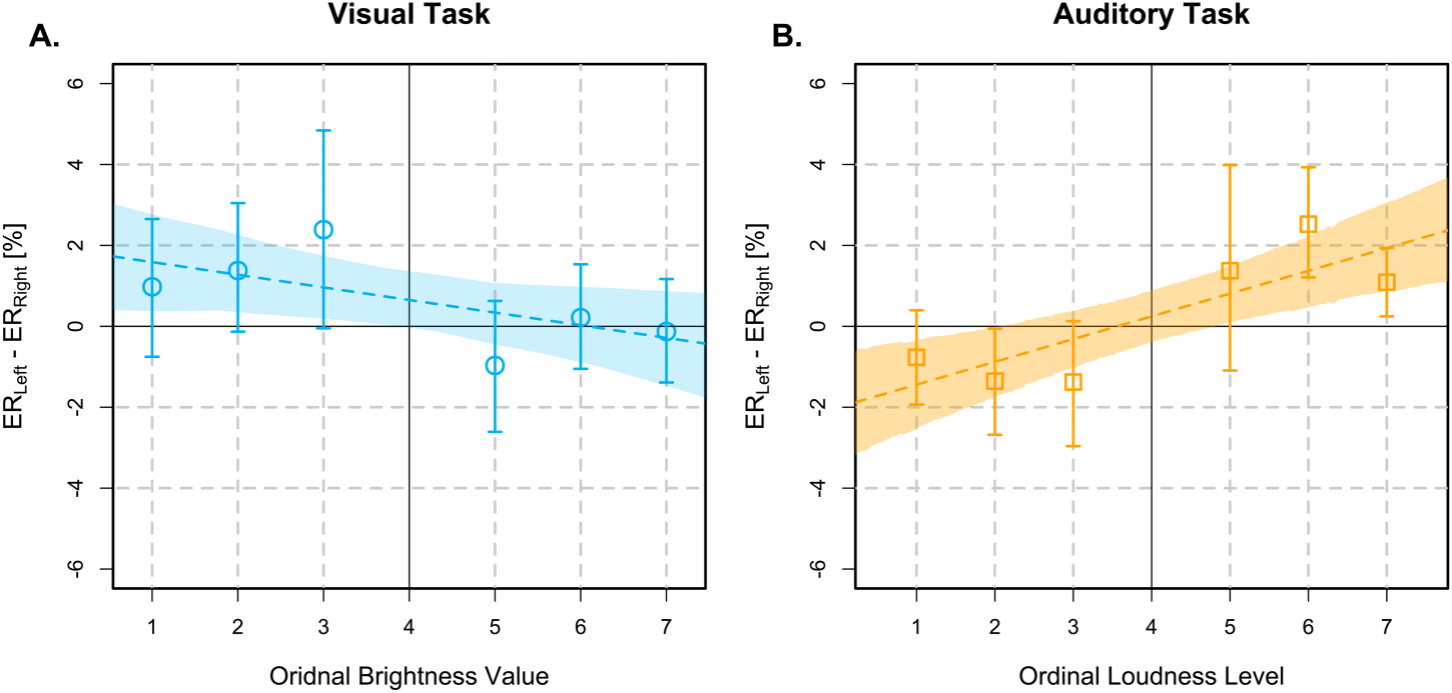
SNARC-like slopes for difference scores in ERs ( ${\text{ER}}_{\text{Left}}-{\text{ER}}_{\text{Right}}$ ) as a function of ordinal stimulus intensity, depicted as blue circles for the brightness discrimination task (Panel A) and orange squares for the loudness discrimination task (Panel B). Error bars represent the 95% HDIs of the difference scores. Dashed lines depict the median posteriors estimates of the Bayesian regression models and shaded regions their 95% HDIs. Abbreviations: SNARC = spatial - numerical association of response codes; ER = error rate; HDIs = highest density intervals.

**Figure 3.**
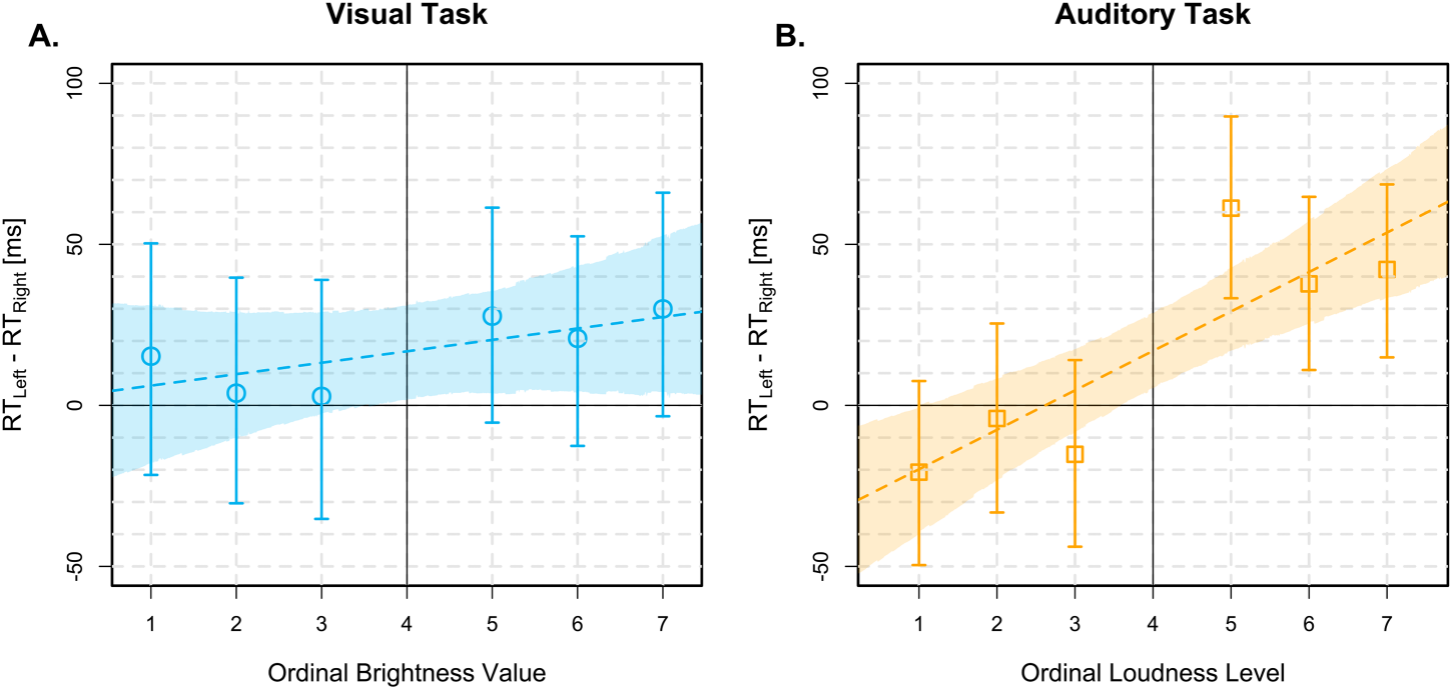
SNARC-like slopes for difference scores in mean correct RTs ( ${\text{RT}}_{\text{Left}}-{\text{RT}}_{\text{Right}}$ ) as a function of ordinal stimulus intensity, depicted as blue circles for the brightness discrimination task (Panel A) and orange squares for the loudness discrimination task (Panel B). Error bars represent the 95% HDIs of the difference scores. Dashed lines depict the median posteriors estimates of the Bayesian regression models and shaded regions are their 95% HDIs. Abbreviations: SNARC = spatial - numerical association of response codes; RTs = response times; HDIs = highest density intervals.

*Loudness discrimination.* Participants made 1.38% fewer errors in the auditory task when the SR-mapping was congruent ( BF=14.09,95%HDI=[−2.25,−0.52] ). The analysis of stimulus-averaged SR-mapping effects on RTs was inconclusive ([ BF=1.51,95%HDI =[−54.90,−3.97] ). Task-averaged RTs and ERs are shown in Figure 1. There was very strong evidence for a positive correlation between ordinal loudness and RT difference scores ( ρ=.18,BF>2,000,95%HDI=[0.10,0.26] ), as well as a strong correlation between loudness and ER difference scores ( ρ=0.15,BF=101.30,95%HDI=[0.07,0.23] ). SNARC-like slopes for ER and RT difference scores are depicted in Figures 2 and 3, respectively.

*Brightness discrimination.* The analysis of ERs in the visual task was inconclusive ( BF=0.55,95%HDI=[−1.8,0.09] ). Similarly, the correlation between brightness and ER difference scores was inconclusive ( ρ=−0.08, BF=0.63,95%HDI=[−0.16,0.00] ). In contrast, there was strong evidence for the null hypothesis for mean RTs, indicating no SR-mapping effect ( BF=0.13,95%HDI=[−41.50,22.06] ). Similarly, the analysis of RT difference scores as a function of ordinal brightness supported the null hypothesis of no correlation ( ρ=0.04, BF=0.17,95%HDI=[−0.04,0.12] ).

**Figure 4.**
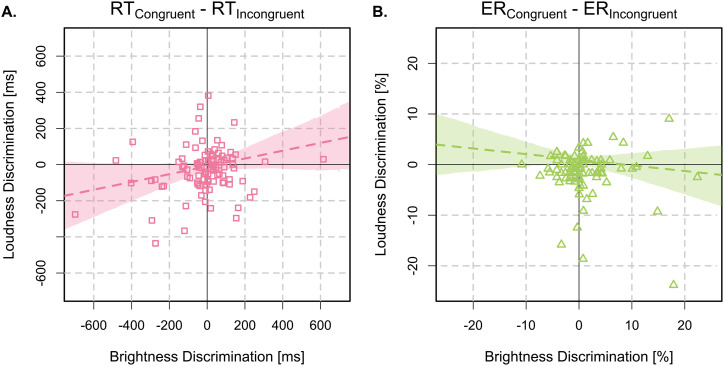
Inter-task correlations between visual and auditory difference scores, plotted separately for mean correct RTs ( ${\text{RT}}_{\text{Congruent}}-{\text{RT}}_{\text{Incongruent}}$ ) as violet squares in Panel A, and ERs ( ${\text{ER}}_{\text{Congruent}}-{\text{ER}}_{\text{Incongruent}}$ ) as green triangles in Panel B. Dashed lines depict the median posterior estimates of the Bayesian regression models, and shaded regions depict the 95% HDIs. Abbreviations: RTs = response times; ER = error rate; HDIs = highest density intervals.

*Inter-task correlations.* The correlation between SR-mapping effects on ERs across the visual and auditory tasks was inconclusive ( ρ=−0.10, BF=0.40,95%HDI=[−0.29,0.09] ). Similarly, the cross-task correlation in RT difference scores was also inconclusive ( ρ=0.18, BF=1.24,95%HDI=[−0.01,0.36] ). These inter-task correlations are illustrated in Figure 4.

## Discussion

This study examined the relationship between SR-mapping effects on human performance in auditory and visual intensity discrimination tasks using laterally separated response keys. Two key questions guided the investigation: (1) whether discrimination performance is influenced by the lateral placement of response keys, in line with a spatial association between stimulus intensity and response codes in these two tasks, and (2) whether this spatial association operates similarly across visual and auditory modalities, as suggested by the polarity correspondence principle and theories of a generalized magnitude system. The following conclusions emerged:

Almost all behavioral indicators of SR association were observed in the loudness discrimination task: congruent SR-mappings resulted in fewer errors, and difference scores in both mean RTs and ERs (reflecting left vs. right response location) were correlated with ordinal stimulus intensity. Although the analysis of stimulus-averaged RTs for congruent versus incongruent mappings was inconclusive, three of the four preregistered hypotheses received strong support. Taken together, these findings strengthen the evidence that speeded loudness judgments of paired auditory stimuli are influenced by lateral response set, aligning with prior reports of such effects (Chang & Cho, 2015; Fairhurst & Deroy, 2017; Hartmann & Mast, 2016).

Notably, the analysis of brightness discrimination data revealed a contrast to loudness discrimination, as none of the preregistered hypotheses were supported, and two could be confidently rejected. This outcome is particularly noteworthy given that our sample size was increased by a factor of ≈3 in relation to previous studies, and the number of measurements per participant by ≈2 . One possible interpretation is that the increased measurement precision in our study helps clarify previously inconsistent findings in the literature, where the direction of lateral space–intensity mapping effects in brightness discrimination has varied across studies (Fumarola et al., 2014; Ren et al., 2011). This interpretation directly challenges the contrast-polarity account often invoked in prior work. Future meta-analyses may help reconcile these divergent results and reveal broader patterns.

An alternative interpretation of these findings is that SR-mapping effects may vary considerably across individuals, reflecting differences in the strength of their learned SR associations. Such variability would not be unexpected, as SR associations in other domains, such as number magnitude, are known to be culturally acquired (e.g., through exposure to left-to-right vs. right-to-left number lines during early education; see Zebian, 2005). Differences in individuals’ experiences may similarly shape spatial associations with intensity; for example, audio engineers might develop strong auditory mappings through frequent interaction with spatially arranged sound controls, whereas individuals in other professions, such as lawyers, may not. Although speculative, this interpretation aligns with the observation that Experiments 1 and 2 by Fumarola et al. (2014) used the same participant pool, potentially mitigating individual differences and increasing the likelihood of conceptual replication.

The absence of detectable correlations between stimulus-averaged SR-mapping effects in RTs and error rates across auditory and visual tasks challenges accounts that posit a shared spatial representation of magnitude across modalities. Combined with the lack of a robust SR-mapping effect in the brightness discrimination task, these findings diverge from predictions based on the polarity correspondence principle and generalized magnitude system theories. However, as noted by Miller and Ulrich (2013), correlations in difference scores are inherently limited by factors such as general and task-specific processing constraints. Consequently, observed cross-task correlations (or their absence) should not be interpreted as direct reflections of the underlying cognitive architecture.

Some limitations of the present research warrant consideration. First, online experiments inherently lack the level of experimental control achievable in laboratory settings. Could this have contributed to some of the null or inconclusive findings? While this possibility cannot be dismissed outright, it cannot fully account for the consistent differences observed between the auditory and visual tasks. Additionally, the feasibility of online psychophysics across common browsers, platforms, and devices used in web experiments have been illustrated (Anwyl-Irvine et al., 2021). A growing body of research also shows that online designs can produce data with acceptable properties (e.g., Germine et al., 2012; Johansson & Ulrich, 2024; Ratcliff & Hendrickson, 2021; Semmelmann & Weigelt, 2017). Indeed, systematic assessments suggest that the most common timing error in online studies is a fixed delay in stimulus presentation or response registration, which tends to shift absolute RT values without introducing substantial variability (Bridges et al., 2020).^5^ Furthermore, RT measurement is generally robust to minor but consistent timing distortions (Ulrich & Giray, 1989). It remains possible, however, that online platforms draw from participant pools that differ in meaningful ways from in-lab populations, complicating direct comparisons with prior reports. While reduced motivation (Jun et al., 2017) and attentiveness (Rodd, 2024) are known concern in remote studies, performance metrics in our sample were within the desired range for all but two participants.

In summary, the present research replicated earlier findings of lateral SR-mapping effects in a paired loudness discrimination task. This reinforces the evidence for a conceptual association between spatial location and perceived loudness—an association reflected in interface design conventions and everyday spatial metaphors for acoustic intensity. In contrast, no comparable effects were observed in the brightness discrimination task: neither RTs nor ERs showed consistent modulation by SR-mapping. Although two of the four analyses yielded only inconclusive evidence for the null hypothesis, the overall pattern points to either modality-specific mechanisms underlying auditory and visual intensity discrimination or considerable individual variability in visual SR-mapping effects that may obscure group-level patterns, even in relatively large samples. Analyses of within-subject correlations across modalities were likewise inconclusive, offering no compelling support for a shared spatial association across tasks. Taken together with the null findings in the visual domain, these results challenge accounts proposing a modality-general left-to-right spatial mapping of perceived intensity. At a minimum, they cast doubt on whether SR-mapping effects in brightness discrimination (and their correspondence with those in audition) are robust or consistent enough to be reliably studied, even with larger-than-standard sample sizes.

## Notes

1. Notably, Ren et al. (2011) found no association between lateral (left–right) response mapping and loudness discrimination performance in a separate study (their Experiment 5).

2. https://osf.io/q6zrh

3. www.prolific.com

4. www.pavlovia.org

5. Audio-visual asynchrony is another serious concern for online studies with bisensory stimuli, but does not directly apply here.

## References

[bibr1-03010066251361080] Anwyl-Irvine A. Dalmaijer E. S. Hodges N. Evershed J. K. (2021). Realistic precision and accuracy of online experiment platforms, web browsers, and devices. Behavior Research Methods, 53, 1407–1425. 10.3758/s13428-020-01501-5 33140376 PMC8367876

[bibr2-03010066251361080] Bridges D. Pitiot A. MacAskill M. R. Peirce J. W. (2020). The timing mega-study: Comparing a range of experiment generators, both lab-based and online. PeerJ, 8, e9414. 10.7717/peerj.9414 PMC7512138 33005482

[bibr3-03010066251361080] Bruzzi E. Talamini F. Priftis K. Grassi M. (2017). A smarc effect for loudness. i-Perception, 8, 204166951774217. 10.1177/2041669517742175 PMC5700794 29201342

[bibr4-03010066251361080] Chang S. Cho Y. (2015). Polarity correspondence effect between loudness and lateralized response set. Frontiers in Psychology, 6, 683. 10.3389/fpsyg.2015.00683 26052305 PMC4440908

[bibr5-03010066251361080] Cohen K. R. Cohen Kadosh K. Henik A. (2008). When brightness counts: The neuronal correlate of numerical-luminance interference. Cerebral Cortex, 18, 337–343. 10.1093/cercor/bhm058 17556772

[bibr6-03010066251361080] Cohen Kadosh R. Henik A. (2006). A common representation for semantic and physical properties: A cognitive-anatomical approach. Experimental Psychology, 53, 87–94. 10.1027/1618-3169.53.2.87 16909932

[bibr7-03010066251361080] Cousineau D. (2005). Confidence intervals in within-subject designs: A simpler solution to Loftus and Masson’s method. Tutorials in Quantitative Methods for Psychology, 1, 42–45. 10.20982/tqmp.01.1.p042

[bibr8-03010066251361080] Dalmaso M. Schnapper Y. Vicovaro M. (2022). When time stands upright: STEARC effects along the vertical axis. Psychological Research, 87, 894–918. 10.1007/s00426-022-01693-9 35718808 PMC10017642

[bibr9-03010066251361080] Dalmaso M. Vicovaro M. (2019). Evidence of SQUARC and distance effects in a weight comparison task. Cognitive Processing, 20, 163–173. 10.1007/s10339-019-00905-2 30721375

[bibr10-03010066251361080] Dehaene S. Bossini S. Giraux P. (1993). The mental representation of parity and number magnitude. Journal of Experimental Psychology, 122, 371–396. 10.1037//0096-3445.122.3.371

[bibr11-03010066251361080] Dehaene S. Dupoux E. Mehler J. (1990). Is numerical comparison digital? Analogical and symbolic effects in two-digit number comparison. Journal of Experimental Psychology: Human Perception and Performance, 16, 626–641. 10.1037/0096-1523.16.3.626 2144576

[bibr12-03010066251361080] Fairhurst M. Deroy O. (2017). Testing the shared spatial representation of magnitude of auditory and visual intensity. Journal of Experimental Psychology: Human Perception and Performance, 43, 629–637. 10.1037/xhp0000332 28240932

[bibr13-03010066251361080] Fumarola A. Prpic V. Da Pos O. Murgia M. Umiltà C. Agostini T. (2014). Automatic spatial association for luminance. Attention, Perception, & Psychophysics, 76, 759–765. 10.3758/s13414-013-0614-y 24402699

[bibr14-03010066251361080] Germine L. Nakayama K. Duchaine B. C. Chabris C. F. Chatterjee G. Wilmer J. B. (2012). Is the web as good as the lab? Comparable performance from web and lab in cognitive/perceptual experiments. Psychonomic Bulletin & Review, 19, 847–857. 10.3758/s13423-012-0296-9 22829343

[bibr15-03010066251361080] Hartmann M. Mast F. (2016). Loudness counts: Interactions between loudness, number magnitude and space. Quarterly Journal of Experimental Psychology, 70, 1–39. 10.1080/17470218.2016.1182194 27109592

[bibr16-03010066251361080] Ishihara M. Keller P. Rossetti Y. Prinz W. (2008). Horizontal spatial representations of time: Evidence for the STEARC effect. Cortex, 44, 454–461. 10.1016/j.cortex.2007.08.010 18387578

[bibr17-03010066251361080] Ito Y. Hatta T. (2004). Spatial structure of quantitative representation of numbers: Evidence from the SNARC effect. Memory & Cognition, 32, 662–73. 10.3758/BF03195857 15478760

[bibr18-03010066251361080] Johansson R. C. G. Ulrich R. (2024). Serial processing of proximity groups and similarity groups. Attention, Perception, & Psychophysics, 86, 1303–1317. 10.3758/s13414-024-02861-2 PMC11093805 38468024

[bibr19-03010066251361080] Jun E. Hsieh G. Reinecke K. (2017). Types of motivation affect study selection, attention, and dropouts in online experiments. Proceedings of the ACM on Human-Computer Interaction, 1, 1–15. 10.1145/3134691

[bibr20-03010066251361080] Koch S. Schubert T. Blankenberger S. (2023). The spatial representation of loudness in a timbre discrimination task. i-Perception, 14, 20416695231213213. 10.1177/20416695231213213 38025962 PMC10652803

[bibr21-03010066251361080] Koch S. Schubert T. Blankenberger S. (2024). Simultaneous but independent spatial associations for pitch and loudness. Psychological Research, 88, 1602–1615. 10.1007/s00426-024-01970-9 38720089 PMC11282129

[bibr22-03010066251361080] Kruschke J. (2014). Doing Bayesian data analysis: A tutorial with R, JAGS, and Stan. Academic Press.

[bibr23-03010066251361080] Lee M. D. Wagenmakers E. J. (2014). Bayesian cognitive modeling: A practical course. Cambridge University Press.

[bibr24-03010066251361080] Lidji P. Kolinsky R. Lochy A. Morais J. (2007). Spatial associations for musical stimuli: A piano in the head? Journal of Experimental Psychology: Human Perception and Performance, 33, 1189–1207. 10.1037/0096-1523.33.5.1189 17924817

[bibr25-03010066251361080] Loftus G. R. Masson M. E. J. (1994). Using confidence intervals in within-subject designs. Psychonomic Bulletin & Review, 1, 476–490. 10.3758/BF03210951 24203555

[bibr26-03010066251361080] Mariconda A. Prpic V. Mingolo S. Sors F. Agostini T. Murgia M. (2022). A systematic investigation reveals that ishihara et al.’s (2008) STEARC effect only emerges when time is directly assessed. Scientific Reports, 12, 18822. 10.1038/s41598-022-23411-6 36335159 PMC9637157

[bibr27-03010066251361080] Miller J. Ulrich R. (2013). Mental chronometry and individual differences: Modeling reliabilities and correlations of reaction time means and effect sizes. Psychonomic Bulletin & Review, 20, 819–858. 10.3758/s13423-013-0404-5 23955122

[bibr28-03010066251361080] Morey R. D. Rouder J. N. (2024) *BayesFactor*. Computation of Bayes factors for common designs [R Package Version 0.9.12-4.7]. 10.32614/CRAN.package.BayesFactor

[bibr29-03010066251361080] Peirce J. Gray J. R. Simpson S. MacAskill M. Höchenberger R. Sogo H. Kastman E. Lindeløv J. K. (2019). PsychoPy2: Experiments in behavior made easy. Behavior Research Methods, 51, 195–203. 10.3758/s13428-018-01193-y 30734206 PMC6420413

[bibr30-03010066251361080] Proctor R. Cho Y. (2006). Polarity correspondence: A general principle for performance of speeded binary classification tasks. Psychological Bulletin, 132, 416–442. 10.1037/0033-2909.132.3.416 16719568

[bibr31-03010066251361080] Ratcliff R. Hendrickson A. T. (2021). Do data from mechanical turk subjects replicate accuracy, response time, and diffusion modeling results? Behavior Research Methods, 53, 2302–2325. 10.3758/s13428-021-01573-x 33825128 PMC8641698

[bibr32-03010066251361080] R Core Team (2021). R: A language and environment for statistical computing. R Foundation for Statistical Computing, Vienna, Austria. https://www.R-project.org/.

[bibr33-03010066251361080] Ren P. Nicholls M. E. R. Ma Y. Chen L. (2011). Size matters: Non-numerical magnitude affects the spatial coding of response. PloS one, 6, e23553. 10.1371/journal.pone.0023553 PMC3154948 21853151

[bibr34-03010066251361080] Rodd J. M. (2024). Moving experimental psychology online: How to obtain high quality data when we can’t see our participants. Journal of Memory and Language, 134, 104472.

[bibr35-03010066251361080] Rusconi E. Kwan B. Giordano B. Umiltà C. Butterworth B. (2006). Spatial representation of pitch height: The SMARC effect. Cognition, 99, 113–129. 10.1016/j.cognition.2005.01.004 15925355

[bibr36-03010066251361080] Semmelmann K. Weigelt S. (2017). Online psychophysics: Reaction time effects in cognitive experiments. Behavior Research Methods, 49, 1241–1260. 10.3758/s13428-016-0783-4 27496171

[bibr37-03010066251361080] Sokolowski H. M. Fias W. Ononye C. B. Ansari D. (2017). Are numbers grounded in a general magnitude processing system? A functional neuroimaging meta-analysis. Neuropsychologia, 105, 50–69. 10.1016/j.neuropsychologia.2017.01.019 28119003

[bibr38-03010066251361080] Stevens S. (1957). On the psychophysical law. Psychological Review, 64, 153–181. 10.1037/h0046162 13441853

[bibr39-03010066251361080] Ulrich R. Giray M. (1989). Time resolution of clocks: Effects on reaction time measurement—Good news for bad clocks. British Journal of Mathematical and Statistical Psychology, 42, 1–12. 10.1111/j.2044-8317.1989.tb01111.x

[bibr40-03010066251361080] Ulrich R. Maienborn C. (2010). Left–right coding of past and future in language: The mental timeline during sentence processing. Cognition, 117, 126–138. 10.1016/j.cognition.2010.08.001 20850112

[bibr41-03010066251361080] Vicovaro M. Dalmaso M. (2021). Is ‘heavy’ up or down? Testing the vertical spatial representation of weight. Psychological Research, 85, 1183–1200. 10.1007/s00426-020-01309-0 32170400

[bibr42-03010066251361080] Vogel S. Grabner R. Schneider M. Siegler R. Ansari D. (2013). Overlapping and distinct brain regions involved in estimating the spatial position of numerical and non-numerical magnitudes: An fMRI study. Neuropsychologia, 51, 979–989. 10.1016/j.neuropsychologia.2013.02.001 23416146

[bibr43-03010066251361080] Walsh V. (2003). A theory of magnitude: Common cortical metrics of time, space and quantity. Trends in Cognitive Sciences, 7, 483–488. 10.1016/j.tics.2003.09.002 14585444

[bibr44-03010066251361080] Winter B. Marghetis T. Matlock T. (2015). Of magnitudes and metaphors: Explaining cognitive interactions between space, time, and number. Cortex, 64, 209–224. 10.1016/j.cortex.2014.10.015 25437376

[bibr45-03010066251361080] Wood G. Willmes K. Nuerk H. C. Fischer M. (2008). On the cognitive link between space and number: A meta-analysis of the SNARC effect. Psychology Science, 50, 489–525. 10.1027/1618-3169.52.3.187

[bibr46-03010066251361080] Zebian S. (2005). Linkages between number concepts, spatial thinking, and directionality of writing: The snarc effect and the reverse snarc effect in English and Arabic monoliterates, biliterates, and illiterate Arabic speakers. Journal of Cognition and Culture, 5, 165–190. 10.1163/1568537054068660

